# Regulation of PI-2b Pilus Expression in Hypervirulent *Streptococcus agalactiae* ST-17 BM110

**DOI:** 10.1371/journal.pone.0169840

**Published:** 2017-01-20

**Authors:** Bruno Périchon, Noémi Szili, Laurence du Merle, Isabelle Rosinski-Chupin, Myriam Gominet, Samuel Bellais, Claire Poyart, Patrick Trieu-Cuot, Shaynoor Dramsi

**Affiliations:** 1 Institut Pasteur, Biology of Gram-positive Pathogens Unit, Paris, France; 2 Centre National de la Recherche Scientifique (CNRS) ERL3526, Paris, France; 3 Institut Pasteur, Unité Ecologie et Evolution de la Résistance aux Antibiotiques, Paris, France; 4 Équipe Barrières et Pathogènes, Institut Cochin, Inserm 1016, CNRS UMR, Paris France; 5 Assistance Publique-Hôpitaux de Paris, Hôpitaux Universitaires Paris Centre-Site Cochin, France; 6 CNR Streptocoques, Hôpitaux Universitaires Paris Centre, site Cochin, AP-HP; 7 DHU Risques et Grossesse, AP-HP, Paris, France; University of Texas Medical School at Houston, UNITED STATES

## Abstract

The widely spread *Streptococcus agalactiae* (also known as Group B *Streptococcus*, GBS) “hypervirulent” ST17 clone is strongly associated with neonatal meningitis. The PI-2b locus is mainly found in ST17 strains but is also present in a few non ST17 human isolates such as the ST-7 prototype strain A909. Here, we analysed the expression of the PI-2b pilus in the ST17 strain BM110 as compared to the non ST17 A909. Comparative genome analyses revealed the presence of a 43-base pair (bp) hairpin-like structure in the upstream region of PI-2b operon in all 26 ST17 genomes, which was absent in the 8 non-ST17 strains carrying the PI-2b locus. Deletion of this 43-bp sequence in strain BM110 resulted in a 3- to 5-fold increased transcription of PI-2b. Characterization of PI-2b promoter region in A909 and BM110 strains was carried out by RNAseq, primer extension, qRT-PCR and transcriptional fusions with *gfp* as reporter gene. Our results indicate the presence of a single promoter (Ppi2b) with a transcriptional start site (TSS) mapped 37 bases upstream of the start codon of the first PI-2b gene. The large operon of 16 genes located upstream of PI-2b codes for the group B carbohydrate (also known as antigen B), a major constituent of the bacterial cell wall. We showed that the hairpin sequence located between antigen B and PI-2b operons is a transcriptional terminator. In A909, increased expression of PI-2b probably results from read-through transcription from antigen B operon. In addition, we showed that an extended 5’ promoter region is required for maximal transcription of *gfp* as a reporter gene in *S*. *agalactiae* from Ppi2b promoter. Gene reporter assays performed in *Lactococcus lactis* strain NZ9000, a related non-pathogenic Gram-positive species, revealed that GBS-specific regulatory factors are required to drive PI-2b transcription. PI-2b expression is up-regulated in the BM110*ΔcovR* mutant as compared to the parental BM110 strain, but this effect is probably indirect. Collectively, our results indicate that PI-2b expression is regulated in GBS ST17 strains, which may confer a selective advantage in the human host either by reducing host immune responses and/or increasing their dissemination potential.

## Introduction

Group B *Streptococcus* (GBS; also known as *Streptococcus agalactiae*) is the leading cause of severe invasive neonatal infections worldwide. Clinical manifestations include pneumonia, septicemia, and meningitis occurring immediately after birth (0–6 days) referred as Early Onset Disease (EOD) or after the 1^st^ week of life (7–90 days) referred as Late Onset Disease (LOD). GBS is a commensal Gram-positive bacterium commonly found in the gastrointestinal and genital tracts of healthy individuals. For EOD, transmission to newborns probably originates from colonized mothers, by inhalation of GBS-contaminated amniotic or vaginal fluid during delivery. For LOD, the mode of transmission and the infection route remain poorly defined.

Several epidemiological studies have pinpointed a remarkable association of serotype III sequence type (ST) 17 GBS strains with meningitis, particularly during LOD [1–3]. These strains belonging to the clonal complex 17 (CC17) have historically been designated as “hypervirulent”. Deciphering the molecular bases of their higher pathogenicity constitute an important focus of our research.

Notably, two specific surface adhesins known as HvgA and Srr2 were shown to enhance the capacity of ST17 strains to cross the blood-brain barrier [4, 5]. In addition, two pilus islands known as PI-1 and PI-2b are found in the majority of ST17 strains [6–8]. Interestingly, a recent analysis of CC17 strains phylogeny using whole genome comparison revealed the loss of pilus island 1 in about 15% of ST17 strains while PI-2b is present in 100% of these strains [9]. Although ubiquitous in the human ST17 lineage, the PI-2b pilus is also found in a few non ST17 human isolates and in most bovine strains [10]. It encodes three pilin subunits (AP1, Spb1, AP2) and two sortases (SrtC1and Srt2) whose functions have been deciphered recently [11]. Two additional genes, *orf* and *lep*, are present at the beginning of the PI-2b operon encoding a conserved hypothetical protein and a putative signal peptidase, respectively. The backbone pilin Spb1 whose crystal structure has been solved recently [12] was proposed to enhance phagocytosis and survival in macrophages [13].

In this work, we carried out a detailed study of PI-2b expression in the ST17 strain BM110 and in the non- ST17 strain A909. We observed a very low expression of PI-2b in BM110 as compared to A909 under laboratory conditions. Comparative genomics indicate the presence of a hairpin structure in the intergenic region upstream from PI-2b in all ST17 strains, which is missing in non- ST17 strains. Deletion of this 43-bp sequence in strain BM110 increased PI-2b transcription by 3- to 5- fold. Lastly, our data suggest a complex regulation of PI-2b expression, being indirectly mediated by CovR and other GBS specific regulatory factors.

## Results

### Expression of major pilin Spb1 is lower in BM110 as compared to A909

**Fig 1:**
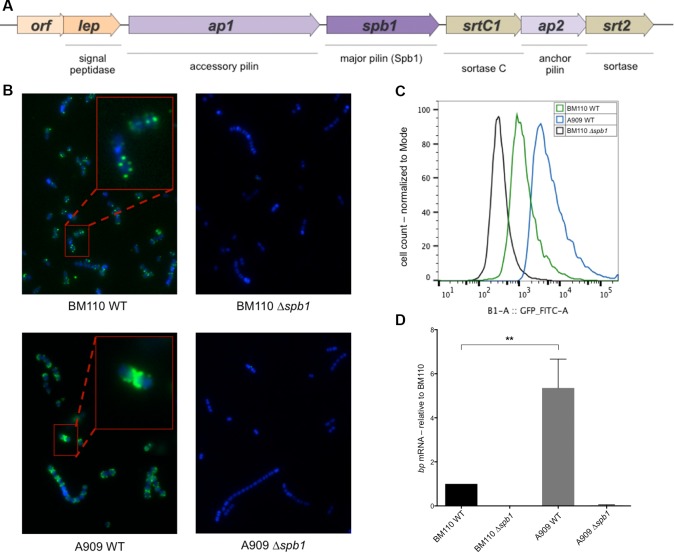
Comparative expression of the gene (*spb1*) encoding the major PI-2b pilin in *S*. *agalactiae* strains BM110 (ST17) and A909 (non ST17). (A) Schematic representation of the PI-2b pilus operon. Arrows represent coding sequence and the direction of transcription. Genes encoding pilus structural proteins are shown in purple, those encoding sortases are in brown, *lep* encodes a putative signal peptidase and *orf* codes for a conserved hypothetical protein. Gene nomenclature is according to [11]. (B) Immunofluorescence microscopy of *S*. *agalactiae* strains using anti-Spb1 antibody. (C) Flow-cytometry analysis of Spb1 expression in WT and isogenic *Δbp* strains. (D) Transcriptional analysis of *spb1* gene by quantitative RT-PCR in exponentially growing *S*. *agalactiae* cells using *gyrA* as an internal standard. Results are expressed as the n-fold change with respect to the WT strain BM110 whose value has been set arbitrarily to 1. Results are means +/- SD from at least two independent cultures in triplicates. Asterisks represent P values (*P<0.05) evaluated using a Student's *t* test.

We previously characterized the PI-2a locus encoding a heterotrimeric pilus in the ST23 strain NEM316 [14, 15]. Like its allelic counterpart, the PI-2b pilus carries 3 genes coding for the structural subunits (*spb1*, *ap1* and *ap2*), 2 genes (*srtC1* and *srt2*) expressing Srt enzymes, and two additional genes (*orf* and *lep*) encoding a small conserved gene of unknown function and a putative signal peptidase, respectively (Fig 1A).

Surface distribution of the major PI-2b pilin Spb1 in strains A909 and BM110 was analysed by immunofluorescence. Spb1 level on the bacterial surface was higher in A909 than in BM110. As shown for other cell-wall anchored proteins, Spb1 preferentially accumulates at the cell poles in A909, whereas in BM110 only 2 to 4 distinct spots *per* bacterium were observed (Fig 1B). As expected, Spb1 protein was undetectable on the surface of A909Δ*spb1* and BM110Δ*spb1* (Fig 1B). Spb1 expression at the single cell level was further characterized using flow-cytometry (Fig 1C). As shown in Fig 1C, Spb1 is expressed homogenously in both strains, and Spb1 level is about 3 to 5 times higher in A909 than in BM110.

Finally, expression of PI-2b was analysed at RNA level by quantitative reverse transcriptase polymerase chain reaction (qRT-PCR) using primers Spb1/Spb2 specific of *spb1* gene (S2 Table). As negative controls, we used the mutants BM110Δ*spb1* and A909Δ*spb1* and the housekeeping *gyrA* gene for normalization. Transcription of *spb1* was about 6-fold higher in A909 as compared to BM110 (Fig 1D).

Collectively, these results demonstrated that expression of Spb1 was higher in A909 than in BM110. This 4- to 6-fold difference in the levels of transcription and surface protein expression suggests a difference in the regulation of PI-2b expression in these two strains.

### Genomic comparison of BM110 and A909 PI-2b locus

**Fig 2:**
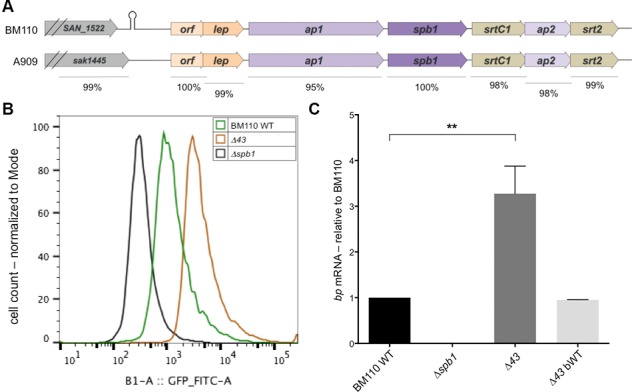
Expression of Spb1 in BM110 and in the isogenic Δ43 mutant (deletion of the 43-bp sequence forming a hairpin-like structure) (A) Schematic comparison of the PI-2b locus in BM110 (ST17 strain) as compared to A909 (non-ST17 strain). (B) Flow-cytometry analysis of Spb1 expression in WT BM110 and isogenic Δ43 mutant, back to wild-type Δ43bWT and Δs*bp1* used as a negative control. (C) Quantitative RT-PCR analysis of RNAs extracted from exponentially growing *S*. *agalactiae*. The expression levels were normalized using *gyrA*. Results are means +/- SD from at least two independent cultures in triplicates. Asterisks represent P values (*P<0.05) evaluated using a Student's *t* test.

Alignment of the PI-2b locus sequence and its immediate genomic environment in BM110 and A909 revealed a strong conservation (Fig 2A). Except for *ap1* encoding the minor pilin of unknown function, the other genes of the PI-2b locus are highly conserved encoding proteins displaying from 98.5 to 100% identity. According to the published A909 genome sequence, AP1 exhibits a premature STOP codon at amino acid 280, leading probably to a non-functional protein. However, re-sequencing of the gene *ap1* (*sak1441*) in strain A909 revealed a missing base at position 839 which suppresses the premature stop codon (our unpublished data). As opposed to the PI-1 and PI-2a pili characterized in GBS, no regulatory gene was found in the vicinity of PI-2b locus (S1 Fig). Immediately upstream from the PI-2b operon lies another large operon of 16 genes (*sak1438*-*1460* in A909) proposed to encode the group B carbohydrate also known as antigen B (AgB) [16].

Comparison of the intergenic region, located between the last gene of the AgB cluster (*sak1445*) and the first gene of the PI-2b locus (*sak1444*), revealed 84% identity between A909 and BM110. The main difference was the presence of a 43-bp sequence in BM110 located at 191 bp upstream from the *orf-lep* segment that was entirely missing in A909 (Fig 2A). This element contains a 17-bp inverted repeat forming a hairpin-like structure which is predicted to be a transcriptional terminator (ΔG = -12.8 kcal/mol) by the ARNold finding terminators web server (http://rna.igmors.u-psud.fr/toolbox/). A similar element of 46-bp (ΔG = -20.04 kcal/mol) was identified as the transcriptional terminator of antigen B in the allelic PI-2a locus of strain NEM316 (S1 Fig).

Analysis of PI-2b intergenic region in 26 ST17 and 8 non-ST17 strains showed that all available ST17 strains possessed this 43-bp sequence, which was absent in all non-ST17 strains.

### Deletion of the 43-bp hairpin increases Spb1 expression in BM110

To test the role of this 43-bp sequence in PI-2b expression, a precise deletion of this element was constructed on the chromosome of WT BM110 strain by homologous recombination in a two-step allelic replacement process. This procedure allowed us to select at the same time the desired mutant BM110Δ43 or an isogenic strain with a wild-type sequence, referred to hereafter as the BM110bWT control strain. In flow-cytometry experiment, the BM110Δ43 mutant showed a 3- to 4-fold increase in Spb1 surface level when compared to the parental BM110 WT (Fig 2B). Western blotting of cell-wall extracts from stationary cultures demonstrated the increased level of pilus polymers in the mutant BM110Δ43 as compared to the BM110 WT or the bWT using specific polyclonal antibodies against the major pilin Spb1. Control strains deleted for *spb1* and for *ap1* were used to show the specificity of the antibodies (S2 Fig).

This difference in protein expression results from a 3- to 4-fold increase in transcription levels of *spb1* and other PI-2b genes (*orf*, *lep*, and *ap1*; data not shown) in the *Δ43* mutant, as revealed by qRT-PCR analysis using specific primers (Fig 2C). The wild type revertant strain (bWT) behaves exactly as the parental wild type BM110.

### Evidence for PI-2b read-through transcription from antigen B operon in GBS A909

**Fig 3:**
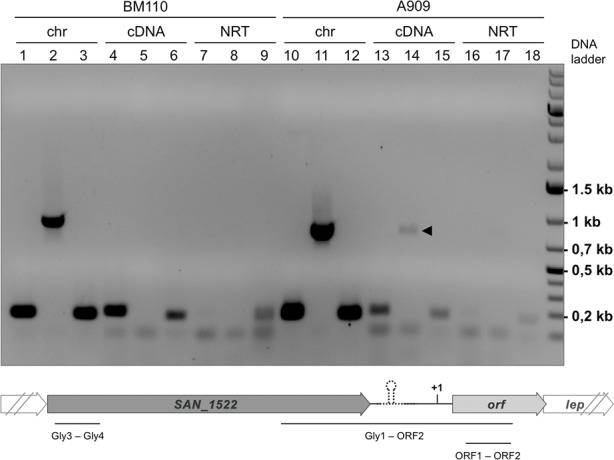
Role of the PI-2b hairpin-like sequence as a transcriptional terminator. Detection of a read-through transcript (arrowhead) encompassing the last gene of AgB operon and the first gene of PI-2b locus in strain A909 but not in strain BM110. RT-PCR was performed using the Gly3/Gly4 (lanes 1, 4, 7, 10, 13, 16), ORF1/ORF2 (lanes 3, 6, 9, 12, 15, 18) and Gly1/ORF2 (lanes 2, 5, 8, 11, 14, 17) oligonucleotides pairs (S2 Table). Lanes 1 to 3 and 10 to 12, positive controls using chromosomal DNA (chr); lanes 4 to 6 and 13 to 15, read-through transcript of *san1522* (according to COH1 annotation) and *orf* (cDNA); Lanes 7 to 9 and 16 to 18 represent negative controls without reverse transcriptase (NRT).

RT-PCR analyses revealed the presence of a read-through transcript encompassing the last gene (*sak1445*) of AgB operon and the first gene (*orf*) of PI-2b locus in strain A909 but not in strain BM110 (Fig 3). This correlates with the absence of a predicted rho-independent terminator sequence in strain A909.

### Mapping of transcription start sites of the PI-2b operon

**Fig 4:**
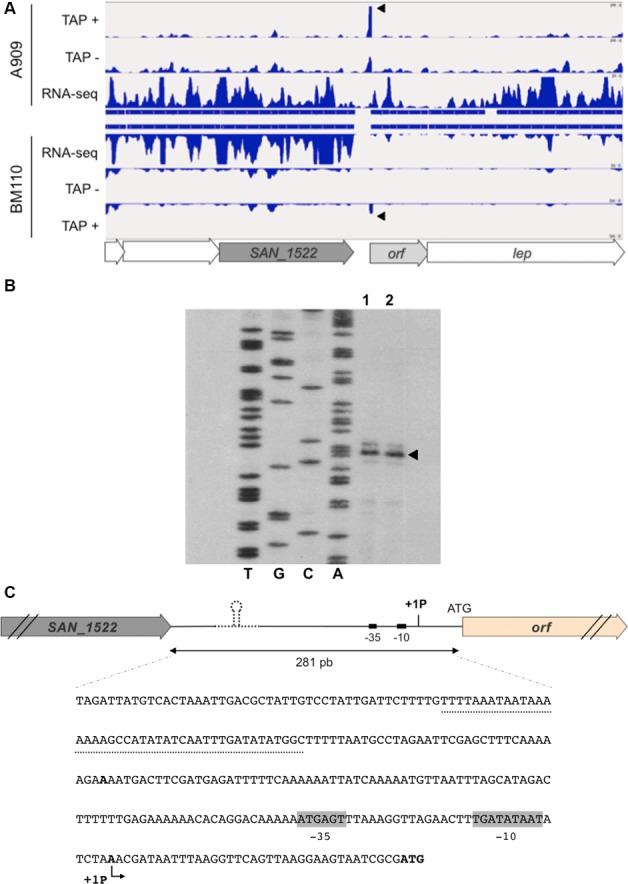
Transcriptional start site of the PI-2b operon. (A) Characterization of *PI-2b* transcription start site in *S*. *agalactiae* strains A909 and BM110 by dRNA-seq. The sequence reads corresponding to transcript 5' ends generated after (TAP+) or without (TAP-) TAP treatment or obtained from strand-specific RNA-seq (RNA-seq) were aligned to the genomes of strains A909 and BM110. A significantly higher number of reads under TAP+ conditions as compared with TAP- is indicative of 5' -triphosphate ends of transcripts characteristic of transcription start sites (TSS). Identical TSS upstream PI-2b locus are detected in strains A909 and BM110. In addition, coverage of the intergenic regions between *sak1445 (or san1522)* and *orf* under conditions of RNA-seq experiments reveals a transcriptional read-through originating from *sak1445*, in A909 only, that could participate to the global level of *PI-2b* transcription in A909. (B) Primer extension analysis of the PI-2b mRNA. Primer elongation product obtained with oligonucleotide E3 and 15 μg of total RNA from A909 (lane1) or BM110 (lane 2). Lanes T, G, C, A, results of sequencing reactions performed with primer E3. Arrow indicated the transcriptional start site. (C) Schematic representation and genomic DNA sequence, from nucleotide positions -281 to +3 (numbering from the A of the ATG start codon of *orf*, negative in the -3'-to-5' direction and positive in the 5' to-3' direction) of the region upstream from the PI-2b locus. Transcription start site is indicated in boldface and arrow. Consensus -10 and -35 sequences are indicated by grey boxes. The 43-bp sequence, present in BM110 and absent in A909, is underlined. The ATG start codon of *orf* is indicated in boldface.

Transcriptional Start Sites (TSS) were identified at the genome level in GBS strains A909 and BM110 using a differential RNA-seq approach as previously described for strain NEM316 [17]. A common TSS was identified 37 bp upstream of the start codon of the first gene (*orf*) of the PI-2b locus in both strains (Fig 4A). In addition, increased transcription was detected in the intergenic region lying between the AgB and PI-2b operons in strain A909, but not in strain BM110 (Fig 4A), which further suggests read-through transcription in strain A909. In contrast a sharp decrease in coverage is observed in strain BM110 upstream from the 43-bp sequence and is in agreement with the proposed role of this hairpin as transcriptional terminator of AgB operon.

Specific mapping of PI-2b TSS in A909 and BM110 was also performed by primer extension analysis. A single signal located 37 bp upstream from the translation initiation codon of *orf* was detected in both strains (Fig 4B). The sequence lying upstream of this TSS displayed a canonical extended -10 (TGATATAAT) and semi-canonical -35 (ATGAGT) boxes separated by 16 bp (Fig 4C).

### A large upstream region is required for maximal transcription of PI-2b in GBS

**Fig 5:**
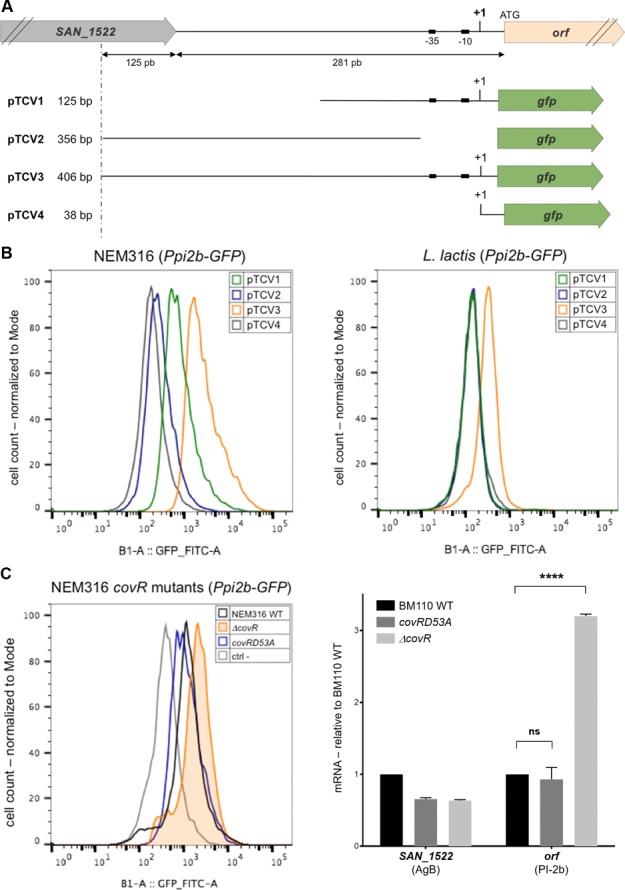
Characterization of PI-2b promoter region using transcriptional fusions with *gfp* as a reporter gene. (A) Schematic representation of the region upstream from the PI-2b locus. The putative +1 transcriptional start site detected by primer extension is indicated. Length of the intergenic region is indicated at the bottom. The four pTCV plasmids (pTCV1 to pTCV4) are drawn schematically. The size of the PI-2b fragment inserted upstream of the reporter gene is indicated on the left. (B) Flow-cytometry analyses of GFP expression in *S*. *agalactiae* NEM316 (ST-23) (left part) or in the heterologous *Lactococcus lactis* strain NZ9000 harboring the various pTCV1-4 plasmids (right part). Left, Black NEM316 + pTCV4; blue, NEM316 + pTCV2; green, NEM316 + pTCV1; orange, NEM316 + pTCV3; Right, Black NZ9000 + pTCV4; blue, NZ9000 + pTCV2; green, NZ9000 + pTCV1; orange, NZ9000 + pTCV3. (C) Role of CovR on PI-2b pilus expression in *S*. *agalactiae*. Left panel, Flow-cytometry analysis of GFP expression in NEM316 WT (black) and isogenic *ΔcovR* (orange) and CovRD53A (blue) strains harboring pTCV3. NEM316 carrying pTCV2 (grey) was used as a negative control. Right panel, transcriptional analysis of *san1522* (AgB locus, according to COH1 annotation) and PI2b-*orf* by quantitative RT-PCR in exponentially growing *S*. *agalactiae* cells using *gyrA* as an internal standard. Results are expressed as the n-fold change with respect to the WT strain BM110 whose value has been set arbitrarily to 1. Results are means +/- SD from at least two independent cultures in triplicates. Asterisks represent P values (****P ≤ 0.001, ns for non- significative) evaluated using a Student's *t* test.

To map precisely the Ppi2b promoter region, fragments encompassing different domains of A909 PI-2b intergenic region (Fig 5A) were cloned upstream from the reporter gene encoding *gfp* using the vector pTCV-GFP, a low-copy number plasmid and assayed for GFP expression in the non-PI-2b GBS strain NEM316 (ST23). The various constructs are drawn schematically on Fig 5A. Plasmid pTCV4 was used as negative control. Quantification of GFP fluorescence was performed using flow-cytometry in exponentially growing bacteria in TH broth supplemented with erythromycin for plasmid maintenance.

A detectable GFP expression was seen with a 125-bp fragment containing the promoter Ppi2B and about 60 bp upstream from the TSS (pTCV1). Higher level of GFP expression was observed with a larger 406-bp fragment including the whole intergenic region of Ppi2b (pTCV3) (Fig 5B). No detectable GFP expression was seen with a fragment encompassing the region upstream without Ppi2b (pTCV2) which indicated that this segment did not contain another uncharacterized promoter.

### GBS specific factors are required for maximal PI-2b transcription

To determine if transcription driven from PI-2b promoter region was dependent on GBS-specific regulatory factors, the various transcriptional fusions (pTCV1 to 4) were introduced into *Lactococcus lactis* strain NZ9000, a non-pathogenic Gram-positive species belonging to the Streptococcaceae family. As shown in Fig 4B, pTCV1 and pTCV2 gave a signal similar to the negative control. A very weak GFP fluorescence activity was measured with the largest promoter fragment (pTCV3). This result suggests that GBS-specific regulatory factors lacking in *L*. *lactis* are required for maximal transcription of PI-2b locus.

CovR was previously shown to be the master regulator of virulence genes in GBS [18, 19]. CovR binds to target DNA sequences when phosphorylated at amino acid D53 by cognate histidine kinase CovS [20]. To assess the contribution of CovR in PI-2b expression, we introduced the pTCV3 *gfp* plasmid in NEM316*ΔcovR* and CovRD53A mutants (Fig 5C). A 2- to 3-fold increase in GFP expression driven from the Ppi2b promoter was consistently observed in NEM316*ΔcovR* as compared to WT NEM316 (Fig 5C). However, this effect was not apparent in CovRD53A mutant (Fig 5C). To test the role of *covR* directly in strain BM110, qRT-PCR analyses comparing mRNA levels of PI-2b first gene (*orf*) and AgB last gene (*san1522*) were carried out in BM110 WT, BM110*ΔcovR* and BM110CovRD53A. As shown in Fig 5C, a 2- to 3- fold increase of PI-2b*orf* was seen in the *ΔcovR*, but not in CovRD53A mutant, as compared to the WT BM110. In contrast, the levels of *san1522*, encoding the last gene of antigen B, appeared unchanged in BM110 WT and *covR* mutants.

Since CovR was shown to directly repress PI-1 pilus transcription in GBS strain 2603V/R [21], we examined PI-1 mRNA levels in BM110 WT, BM110*ΔcovR* and BM110 D53A by qRT-PCR. In agreement with Jiang results, we showed that transcription of the gene *san0698* encoding the PI-1 major pilin in BM110 increased 2.5-fold in the *ΔcovR* strain and 5-fold in the CovRD53A mutant (S3 Fig). Collectively, these results demonstrate a direct role of CovR in PI-1 regulation in GBS strain BM110 and suggest an indirect role of CovR in PI-2b expression.

## Discussion

Pili are important virulence factors for many Gram-positive bacteria. In *S*. *agalactiae*, three different pilus loci have been described, PI-1, PI-2a and PI-2b, the latter two being mutually exclusive allelic variants [14, 22]. The PI-1 locus is present on a mobile genetic element while PI-2 is located on the core genome [7, 23]. The PI-2b pilus was primarily associated with the epidemiologically relevant clinical isolates belonging to CC17, but was not strictly restricted to this “hypervirulent” lineage causing the majority of neonatal invasive diseases. Two genomic studies investigating several contemporary CC17 isolates from Canada and South China demonstrated conservation of the PI-2b locus but loss of the PI-1 pilus which is replaced by novel mobile genetic elements encoding determinants of antimicrobial resistance [9, 24]. Unlike the other two pilus loci PI-1 and PI-2a, no regulatory gene was found in the vicinity of PI-2b and the regulation of PI-2b expression has not been studied so far. It is worth mentioning that several animal isolates possess a highly similar PI-2b locus, including most bovine strains, and several fish isolates [25].

In this work, we aimed at deciphering the molecular bases of PI-2b regulation to uncover the possible role of PI-2b in the pathophysiology of ST17 strains. Comparison of PI-2b expression between the ST17 human clinical isolate BM110 and a non-ST17 human pathogen A909 revealed a 5-fold higher level of PI-2b transcripts in strain A909, which translates into a 5-fold increase of PI-2b major pilin on bacterial surface. The PI-2b locus is highly similar in these two strains, apart from a 43-bp sequence forming a 35-bp hairpin structure located in the intergenic region, 184 bp upstream from the first PI-2b gene. This predicted hairpin can be found in all of the available genome in the NCBI database of ST17 isolates while missing from all non-ST17 isolates that carry the PI-2b locus, suggesting a conserved regulatory role in ST17 strains. Immediately upstream from PI-2b pilus locus lies the putative operon of 16 genes encoding the group B carbohydrate (also known as antigen B) as predicted by a bioinformatic analysis [16]. RNA-seq and qRT-PCR analyses indicate that the hairpin found between the loci encoding antigen B and PI-2b can serve as a transcriptional terminator in strain BM110, and its absence in A909 results in read-through transcripts. A single TSS, located 37 bp upstream from the start codon of the first PI-2b gene (*orf*), exhibiting close-to-canonic -10 and -35 boxes was identified in both GBS strains A909 and BM110 using RNA-seq and primer extension analyses.

The biological relevance of this Ppi2b promoter in PI-2b transcription was assessed at both transcriptional and protein levels using reporter plasmids carrying *gfp* as a reporter gene. The various constructs containing or not Ppi2b were cloned upstream from the reporter gene and expressed in the non-PI-2b GBS strain NEM316. Our results showed that the entire AgB-PI-2b intergenic region is needed for maximal GFP transcription. A smaller region containing Ppi2b was also able to promote GFP expression albeit at lower levels than with the entire upstream region. Expression of these reporter constructs in *L*. *lactis* strain NZ9000, a related non-pathogenic Gram-positive species, revealed that GBS-specific regulatory factors are required to drive PI-2b transcription. Only a very weak gene reporter expression was observed in *L*. *lactis* NZ9000 using the entire PI-2b promoter region. No GFP activity could be detected with any of the reporter constructs in *L*. *lactis* at 30°C, although it is the optimal temperature for *L*. *lactis* growth and for GFP folding (data not shown). In GBS, the main regulator of virulence genes is CovR, the response regulator of the two-component system known as CovRS (or CsrRS) [18, 19]. CovS phosphorylates CovR on aspartyl residue D53, which in turn binds to target promoters (i.e. *cyl* operon) and repress gene expression in most cases. Thus, a CovRD53A mutant, which cannot be phosphorylated, must behave like a *ΔcovR* mutant. Genetic analyses indicate that CovR represses the expression of both PI-1 and PI-2b pili in GBS strain BM110. CovR repression on PI-1 is strong (10-fold difference) and direct (de-repression in the punctual mutant CovRD53A) whereas the effect on PI-2b is only 2-fold and probably not direct. These results are consistent with recent data showing that CovR directly repress the expression of PI-1 pilus in strain 2603V/R [21].

The overall effect of the 43-bp hairpin presence, shown to be a transcriptional terminator, is to reduce PI-2b expression on the bacterial surface of ST17 strains as compared to non-ST17 strains, which include one human A909 and several bovine isolates in our collection [26]. We speculate that low levels of PI-2b convey a selective advantage to ST17 invasive isolates in a human host most likely by reducing host immune responses and/or increasing the dissemination potential of ST17 strains. In line with the second hypothesis, approximately 90% of the weak biofilm producers belonged to CC17 and CC19 which are responsible for the majority of neonatal and adult invasive infections respectively [25]. Interestingly, bovine strains were found to form much stronger biofilms when compared to the human strains. It is thus tempting to speculate that high expression of PI-2b locus in bovine strains could contribute to biofilm formation and that this increased colonization is associated to commensalism state.

We hypothesize that the presence of the 43-bp sequence forming a hairpin-like structure and its negative effect on PI-2b expression is part of the host-adaptation of the hypervirulent ST17 lineage. Future research will focus on identifying environmental signals or host specific cues modulating PI-2b expression in ST17 strains.

## Material and Methods

### Bacterial strains, plasmids, and growth conditions

The bacterial strains and plasmids used in this study are listed in S1 Table. Primers are listed in S2 Table. GBS ST17 clinical strains isolated from invasive infections (i.e. from blood culture, cerebro-spinal fluid or other sterile sites) were obtained from the French National Reference Center for Streptococci in Paris (http://www.cnr-strep.fr) [5]. GBS NEM316, capsular serotype III ST-23, A909, capsular serotype Ia ST-9 and GBS BM110 capsular serotype III ST17 are well-characterized isolates from human with invasive infections. Seven non-ST17 GBS isolates (HN016, WC135, YM001, GX064, LMG14608, LMG15083, BSU178) whose genome are available were previously described [10, 26]. *Escherichia coli* DH5α (Gibco-BRL) was used for cloning experiments.

*S*. *agalactiae* strains were cultured in Todd Hewitt (TH) broth or agar (Difco Laboratories, Detroit, MI) at 37°C in standing filled flasks and *E*. *coli* in Luria-Bertani (LB) medium. *Lactococcus lactis* NZ9000 was grown in M17 medium supplemented with 1% glucose at 30°C or 37°C. Antibiotics were used at the following concentrations: for *E*. *coli*, erythromycin, 150 μg ml^−1^, kanamycin, 50 μg ml^−1^; for *S*. *agalactiae*, erythromycin, 10 μg; for *L*. *lactis*, erythromycin, 5 μg ml^−1^.

### General DNA techniques

Standard recombinant techniques were used for nucleic acid cloning and restriction analysis. Plasmid DNA from *E*. *coli* was prepared by rapid alkaline lysis using the Qiaprep Spin Miniprep kit (Qiagen). Genomic DNA from *S*. *agalactiae* was prepared using the DNeasy Blood and Tissue kit (Qiagen). PCR was carried out with Phusion Taq polymerase as described by the manufacturer (Finnzymes). Amplification products were purified with QIAquick PCR purification kit (Qiagen) and verified by sequencing.

### Real-time quantitative PCR

Bacterial strains pre-cultured overnight in TH broth at 37°C in standing filled cultures were diluted to OD_600_ = 0.05 in 25 ml TH and grown at 37°C until early exponential growth phase (OD_600_ = 0.3). After centrifugation of 20 mL (4°C, 5,200 g), total RNA extraction was performed using the FastRNA PRO BLUE Kit (MP Biomedicals). RNA quality was assessed by agarose gel electrophoresis, residual DNA from the RNA samples was removed with the TURBO DNase kit (2U/μl, Ambion / Thermo Fischer Scientific) and extracts were stored at -80°C. RNA concentrations were measured using Nanodrop 2000c (Thermo Fischer Price). Reverse transcription (RT) was performed with the iScript cDNA synthesis kit (Bio-Rad). Specific primer pairs were designed to obtain a predicted amplicon size of 170 to 220 bp (see S2 Table) and quantitative PCR (qPCR) was carried out with EvaGreen Universal qPCR Supermix (Bio-Rad) in a CFX96 Touch Real-Time PCR Detection System (Bio-Rad). Relative gene expression levels were calculated with the ΔΔCq method [27] where expression values were normalized with the expression of the housekeeping gene *gyrA* and to a control sample by the CFX Manager Software v3.0 (Bio-Rad). Each assay was performed in triplicate with three independent cultures.

### Primer extension reactions

Total RNA was used as template for primer extension reaction using a radiolabeled specific primer complementary to a sequence located downstream from the putative PI-2b promoter 2bEA3 (S2 Table). For detail, synthetic oligodeoxynucleotides were 5'-end-labelled with [γ-^32^P]-ATP (110 TBq mmol^-1^) using T4 polynucleotidekinase. 15 μg of RNA and 2 pmol of labelled oligo-nucleotide were annealed in a total volume of 18 μl of reverse transcriptase buffer (50 mM Tris-HCl; 8 mM MgCl_2_; 30 mM KCl; 1mM DTT; pH 8.5). The mixture was incubated for 3 min at 65°C and then 1 μl (25 U) of avian myelo-blastosis virus (AMV, Boehringer) reverse transcriptase and 1μl of all dNTPs mix (20 mM each) were added. After 30 min at 42°C, reactions were stopped by the addition of 5 μl of a solution containing 97.5% deionized formamide, 10 mM EDTA, 0.3% xylene cyanol, and 3.3% bromo-phenol blue.

The corresponding Sanger DNA sequencing reactions were carried out by using the same primer and a PCR-amplified fragment containing the PI-2b upstream region (primer pair 2bEA1 and 2bEA2, S2 Table) with the Sequenase PCR product sequencing kit (USB).

### Genome-wide mapping of transcription start sites (TSSs)

Genome-wide mapping of transcription start sites in GBS strains A909 and BM110 was performed by using a differential RNA-sequencing protocol based on selective Tobacco Acid Pyrophosphatase (TAP) treatment and 5' adapter ligation as previously described [17, 28]. Briefly, for each strain, mixes of RNA prepared at mid-exponential and stationary growth phases in a rich culture medium (TH) and at the beginning of the stationary phase in a poor culture medium (RPMI supplemented with glucose 1% and pH-buffered with 50 mM Hepes) were enriched in mRNA with the MICROBExpress Kit (Ambion). dRNA-seq libraries were prepared after or without prior TAP treatment by ligation with a 5' adapter (Illumina TruSeq Small RNA kit) and by reverse transcription using a random primer. After sequencing the reads were aligned to the reference genomes (A909: NC_007432.1; BM110: unpublished complete genome sequence obtained from PacBio sequencing) and analyzed for statistical assignment of TSSs based on the differences between the number of reads originating at each sequence position under TAP+ and TAP- conditions. In addition, strand-specific RNA-seq libraries were prepared from the two strains grown at mid-exponential growth phase by using the Illumina primer ligation method. dRNA-seq and RNA-seq libraries were sequenced on the Illumina GAIIX or HiSeq 2000. Alignments of the reads to the reference genomes were visualized with the IGV genome browser [29].

### Construction of mutants

In frame deletion mutants of *spb1* in BM110 and A909 were constructed as previously described [13]. The deletion was confirmed by PCR and sequencing on the genomic DNA of the mutants.

### Generation of polyclonal antibody

DNA fragment intragenic to *spb1* (from amino acids 26–460) was amplified by PCR using genomic DNA of GBS BM110 as a template and primers listed in S2 Table. The amplification product was cloned into the pGEM-T easy vector (TOPO Zero Blunt for Ap1). After verification by sequencing, the fragment was digested with the appropriate enzymes and cloned into pET28a. The resulting plasmid was introduced into *E*. *coli* BL21λDE3/pDIA17 for protein expression. Recombinant proteins were purified on Ni-NTA columns. Protein purity was checked on sodium dodecyl sulfate polyacrylamide gel electrophoresis (SDS-PAGE), and accurate protein concentration was determined using the Bradford protein assay. Rabbits polyclonal antibodies were produced and purchased from Covalab.

### Immunofluorescence microscopy

GBS strains A909 and BM110 were grown overnight in Todd-Hewitt broth. Bacteria were washed twice in phosphate buffered saline (PBS), and incubated for 1h at 4°C with rabbit primary antibody anti-Spb1 diluted in PBS-BSA 1.5% (1/1000). After two washes with PBS, samples were incubated for 30 min with secondary DyLight_488_-conjugated goat anti-rabbit immunoglobulin diluted in PBS-BSA 1.5% (1/1000 dilution; Thermo Scientific Pierce) and Hoechst 33342 (1/1000). Microscopic observations were done with a Nikon Eclipse Ni-U and images acquired with a Nikon Digital Camera DS-U3. Images taken at different color channels were treated and merged in Image J.

### Flow-cytometry

To analyze PI-2b expression, exponentially growing bacteria (OD_600_ = 0.3–0.4), were collected and washed twice in PBS. Bacterial pellet was incubated with rabbit anti-Spb1 primary antibody (1/1000) diluted in PBS-BSA 1% for 1 h at 4°C. After two washes with PBS, samples were incubated with the secondary antibody DyLight_488_-conjugated goat anti-rabbit immunoglobulin (Thermo Scientific Pierce) diluted 1/1000 in PBS-BSA 1% for 30 min at 4°C. *S*. *agalactiae* and *L*. *lactis* strains carrying GFP reporter constructs were grown overnight at 30°C in 2 ml TH broth or M17-glucose, respectively, diluted 20x the following day, and grown at 37°C to exponential phase. One ml samples were harvested, and Hoechst 33342 was directly added in the medium (1/1000), the tubes were left open and incubated 10 minutes under gentle agitation. After washing and resuspension in PBS, samples were acquired on a MACSQuant YGV Analyzer apparatus (Miltenyi Biotec) and data were analyzed using FlowJo X software.

### Cell wall protein extraction

Bacteria were grown in TH medium at 37°C and harvested for protein analysis during late exponential phase of culture. Bacteria were washed in PBS and then resuspended in mutanolysin digestion mix (100Uml^-1^ Mutanolysin (Sigma) resuspended in 50 mM Tris-HCl, pH 7.3, 20% sucrose, supplemented with 1x Proteinase Inhibitor Complex (Roche). The digestion was performed for 2 h at 37°C under gentle agitation. After centrifuging at 13,000 g for 10 min at 4°C, supernatants corresponding to the cell wall fractions were analyzed on SDS-PAGE or kept frozen at −20°C.

### Western immunoblots

For analysis of major- and ancillary- pilin expression, cell wall proteins were boiled in Laemmli sample buffer, separated by SDS-PAGE on 4–12% Tris-Acetate Midi Criterion XT Precast gels (Bio-Rad), and transferred to PVDF membrane using the Trans-Blot Turbo transfer pack (Bio-Rad). Immuno-detection was performed as follow: membrane was blocked in TBS–skimmed milk 5% and incubated for 1 h with rabbit primary Spb1 and Ap1 antibodies and then with the secondary Dylight_800_-coupled goat anti-rabbit antibody (Thermo Scientific Pierce). Between the two antibodies and before detection, membranes were extensively washed with TBS + 0.1% Tween 20. Bound pilins were detected using the LI-COR Odyssey Infrared Imaging System (LI-COR Biosciences).

### Statistical analysis

Data were analyzed using GraphPad Prism 6.0 (GraphPad Software, San Diego, California). The significance of differences between the values was determined by Student’s t-test. Significance levels were set at *P ≤ 0.05; **P ≤ 0.01; ***P ≤ 0.005; ****P ≤ 0.001.

## Supporting Information

S1 Fig Schematic comparison of the PI-2a genomic locus in strain NEM316 versus the allelic PI-2b locus in strain A909. The gene annotated *gbs1479*, also known as *rogB* [30], encoding a RofA-like transcriptional regulator, is shown in green. No regulatory gene can be found in the vicinity of PI-2b operon.
(TIF)

S2 Fig Expression of the PI-2b major (Spb1) and minor (Ap1) pilins in *S*. *agalactiae* strains BM110 (ST-17) and A909 (non ST-17). Western blot analysis of cell wall anchored proteins isolated from *S*. *agalactiae* BM110 and A909, separated on 4%-12% gradient Criterion XT SDS-PAGE, and detected by immunoblotting with specific polyclonal anti-Spb1 and anti-Ap1 antibodies. Equivalent amounts (15 μg) of total protein was loaded in each well. The monomers (m) and high-molecular weight species corresponding to pili polymers (p) of Spb1 and Ap1 are indicated; (d) means degradation products.
(TIF)

S3 Fig Role of CovR on PI-1 pilus expression in *S*. *agalactiae* BM110. Transcriptional analysis of *san0698* (according to COH1) encoding the major pilin of the PI-1 pilus by quantitative RT-PCR in exponentially growing *S*. *agalactiae* cells using *gyrA* as an internal standard. Results are expressed as the n-fold change with respect to the WT strain BM110 whose value has been set arbitrarily to 1. Results are means +/- SD from at least two independent cultures in triplicates. Asterisks represent P values (****P ≤ 0.001, ns for non-significant) evaluated using a Student's *t* test.
(TIF)

S1 File Additional references. (DOCX)

S1 Table Strains and plasmids used in this study. (DOCX)

S2 Table Primers used in this study. (DOCX)
