# Supplementary figures and images for: Regulation of PI-2b Pilus Expression in Hypervirulent Streptococcus agalactiae ST-17 BM110
Source: PLoS One. 2017 Jan 20;12(1):e0169840. doi: 10.1371/journal.pone.0169840 (PMC5249243; doi:10.1371/journal.pone.0169840)

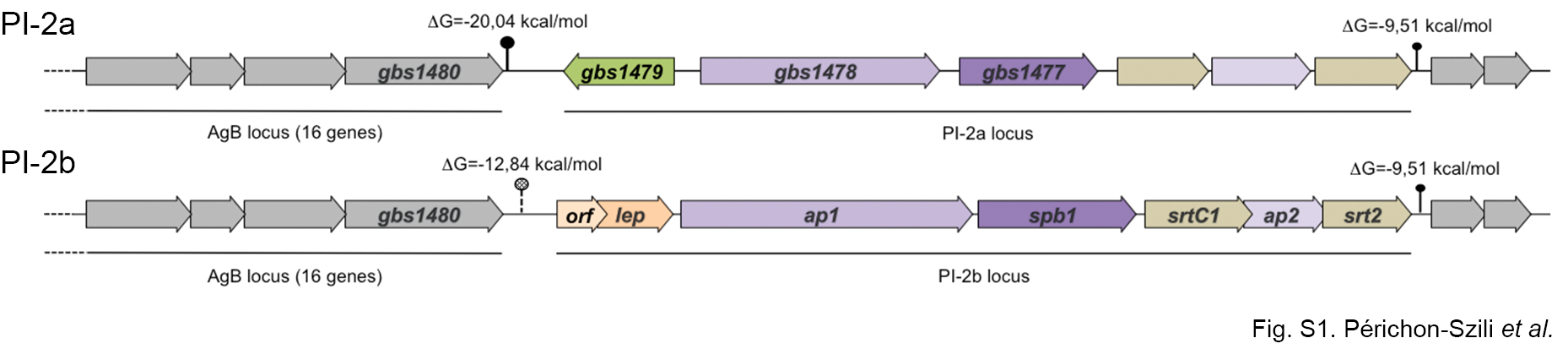

Supplement: S1 Fig — The gene annotated gbs1479, also known as rogB [30], encoding a RofA-like transcriptional regulator, is shown in green. No regulatory gene can be found in the vicinity of PI-2b operon. (TIF) [file pone.0169840.s001.tif]

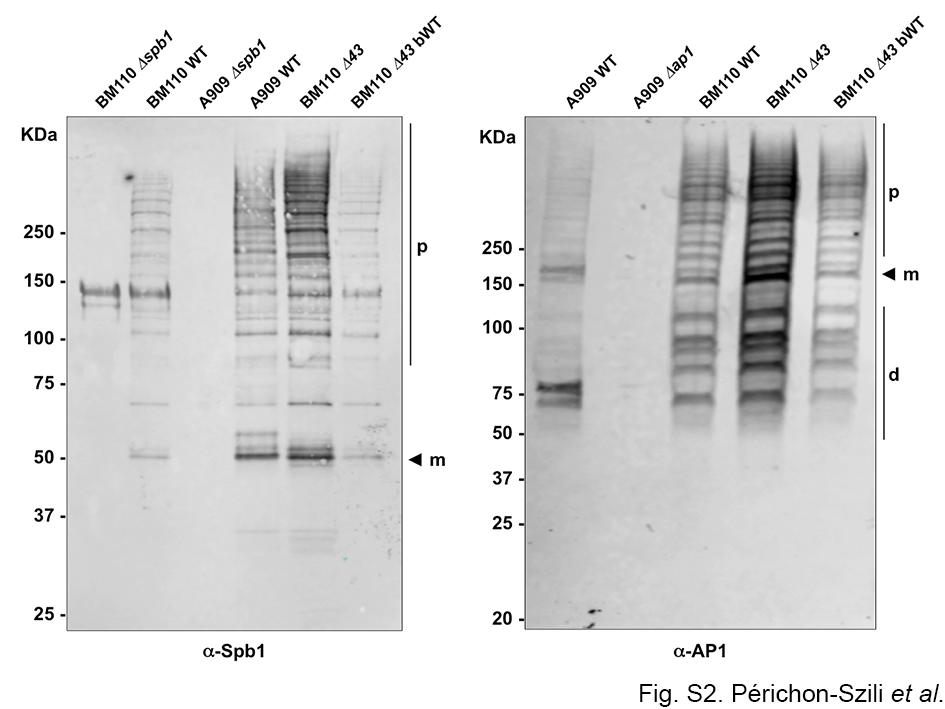

Supplement: S2 Fig — Western blot analysis of cell wall anchored proteins isolated from S. agalactiae BM110 and A909, separated on 4%-12% gradient Criterion XT SDS-PAGE, and detected by immunoblotting with specific polyclonal anti-Spb1 and anti-Ap1 antibodies. Equivalent amounts (15 μg) of total protein was loaded in each well. The monomers (m) and high-molecular weight species corresponding to pili polymers (p) of Spb1 and Ap1 are indicated; (d) means degradation products. (TIF) [file pone.0169840.s002.tif]

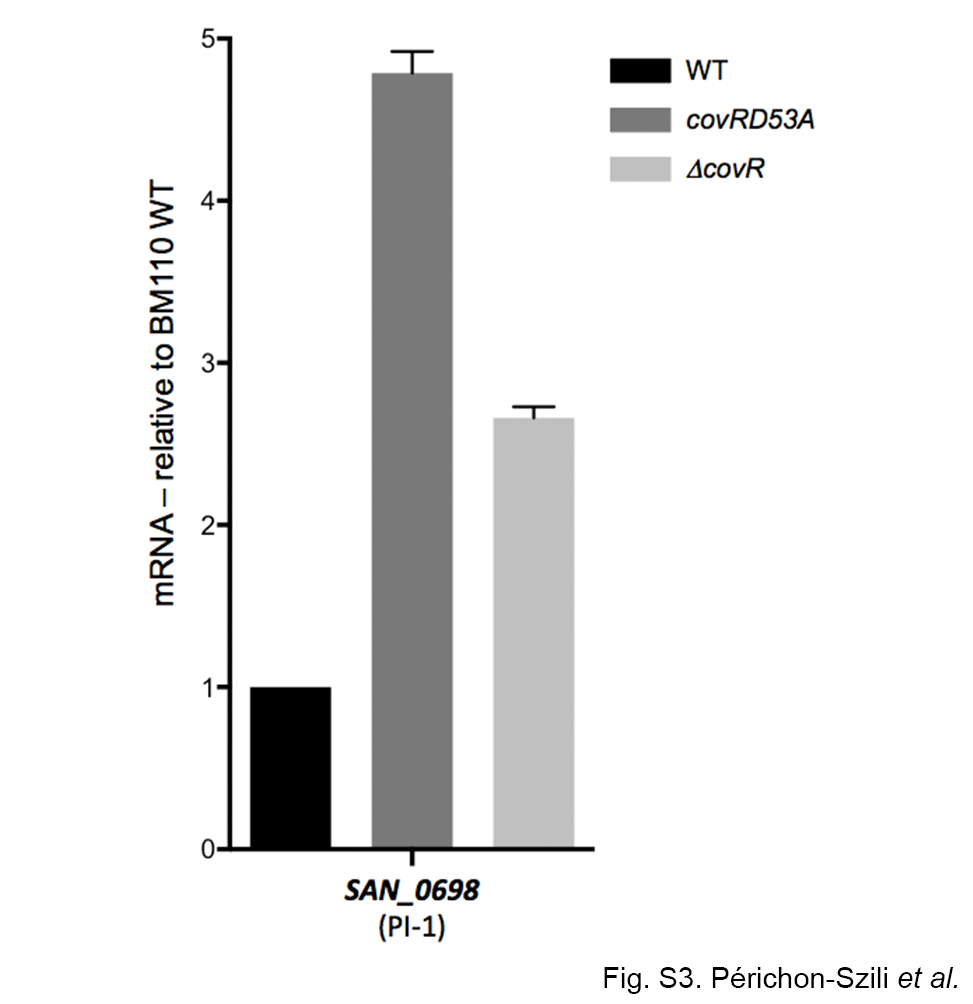

Supplement: S3 Fig — Transcriptional analysis of san0698 (according to COH1) encoding the major pilin of the PI-1 pilus by quantitative RT-PCR in exponentially growing S. agalactiae cells using gyrA as an internal standard. Results are expressed as the n-fold change with respect to the WT strain BM110 whose value has been set arbitrarily to 1. Results are means +/- SD from at least two independent cultures in triplicates. Asterisks represent P values (****P ≤ 0.001, ns for non-significant) evaluated using a Student's t test. (TIF) [file pone.0169840.s003.tif]
