# Supplementary material for: Regulation of PI-2b Pilus Expression in Hypervirulent Streptococcus agalactiae ST-17 BM110
Source: PLoS One. 2017 Jan 20;12(1):e0169840. doi: 10.1371/journal.pone.0169840 (PMC5249243; doi:10.1371/journal.pone.0169840)
Supplement: S1 File — (DOCX) [file pone.0169840.s004.docx]

**Supporting Information**

**Additional references**

30. Lindahl G, Stålhammar-Carlemalm M, Areschoug T (2005) Surface proteins of *Streptococcus agalactiae* and related proteins in other bacterial pathogens. Clin Microbiol Rev 18: 102-127.

31. Lancefield RC, McCarty M, Everly WN (1975) Multiple mouse-protective antibodies directed against group B streptococci. Special reference to antibodies effective against protein antigens. J Exp Med 142: 165-179.

32. Glaser P, Rusniok C, Buchrieser C, Chevalier F, Frangeul L, Msadek T, et al. (2002) Genome sequence of *Streptococcus agalactiae*, a pathogen causing invasive neonatal disease. Mol Microbiol 45: 1499-1513.

33. Firon A, Tazi A, Da Cunha V, Brinster S, Sauvage E, Dramsi S, et al. (2013) The Abi-domain protein Abx1 interacts with the CovS histidine kinase to control virulence gene expression in group B streptococcus. PLoS Pathog 9: e1003179

34. Kuipers OP, de Ruyter PG, Kleerebezem M, de Vos WM (1998) Quorum sensing-controlled gene expression in lactic acid bacteria. J Biotechnol 64: 15-21
