# Supplementary material for: Regulation of PI-2b Pilus Expression in Hypervirulent Streptococcus agalactiae ST-17 BM110
Source: PLoS One. 2017 Jan 20;12(1):e0169840. doi: 10.1371/journal.pone.0169840 (PMC5249243; doi:10.1371/journal.pone.0169840)
Supplement: S1 Table — (DOCX) [file pone.0169840.s005.docx]

**S1 Table. Strains and plasmids used in this study**

| Strain or plasmid | Relevant characteristics | Reference |
| --- | --- | --- |
| Strains |  |  |
| *E. coli* |  |  |
| DH5α™ | F– Φ80Δl*acZ*Δ*M15* Δ(*lacZYA*-*argF*) U169 *recA1* *endA1* *hsdR17* (rK–, mK+) *phoA* supE44 λ– *thi-1* *gyrA96* *relA1* | Invitrogen™ |
| *S. agalactiae* |  |  |
| BM110 | Serotype III, ST-17, human clinical isolate | [30] |
| BM110Δ*covR* | In frame deletion of *covR* in BM110 | Asma Tazi |
| BM110CovRD53A | BM110 mutant expressing CovR with a D53A substitution | Arnaud Firon |
| BM110*∆bp* | In frame deletion of *sbp1* (*san1519 according to COH1*) | This study |
| BM110*∆bp* bWT | Back to the WT strain obtained during the construction of BM110*∆bp* | This study |
| BM110*∆43* | Deletion of the 43-bp sequence  (5' GTTTTAAATAATAAAAAAAGCCATATATCAATTTGATATATGGC) | This study |
| BM110*∆43* bWT | Back to the WT strain obtained during the construction of BM110*∆43* | This study |
| A909 | Serotype Ia, ST-7, human clinical isolate | [31] |
| A909*∆bp* | In frame deletion of *bp* (*sak1439*) | This study |
| A909*∆bp* bWT | Back to the WT strain obtained during the construction of A909*∆bp* | This study |
| NEM316 | Serotype III, ST-23, human clinical isolate | [32] |
| NEM316Δ*covR* | In frame deletion of *covR* in NEM316 | [33] |
| NEM316CovRD53A | NEM316 mutant expressing CovR with a D53A substitution | [33] |
| *L. lactis* |  |  |
| NZ9000 | *L. lactis* subsp. *cremoris* MG1363 containing the *nisRK* gene in the genome | [34] |
| Plasmids |  |  |
| pTCVΩ*gfp* | *EGFP* expression vector |  |
| pTCV1 | pTCVΩ*gfp* with 125-bp PCR product ( 2bUp14^a^ / 2bUp12) from A909^b^ | This study |
| pTCV2 | pTCVΩ*gfp* with 356-bp PCR product ( 2bUp6 / 2bUp20) from A909 | This study |
| pTCV3 | pTCVΩ*gfp* with 406-bp PCR product with 2bUp6 / 2bUp12 from A909 | This study |
| pTCV4 | pTCVΩ*gfp* with 38-bp DNA fragment (2bUp1/2bUp2) (AAACGATAATTTAAGGTTCAGTTAAGGAAGTAATCGCG) | This study |

^a^ Primer sequences are listed in Table S2.

^b^ Genomic DNA of the corresponding strain was used as template for sequence amplification
