# Supplementary material for: Regulation of PI-2b Pilus Expression in Hypervirulent Streptococcus agalactiae ST-17 BM110
Source: PLoS One. 2017 Jan 20;12(1):e0169840. doi: 10.1371/journal.pone.0169840 (PMC5249243; doi:10.1371/journal.pone.0169840)
Supplement: S2 Table — (DOCX) [file pone.0169840.s006.docx]

**S2 Table. Primers used in this study**

| Primer | Sequence (5'-3') |
| --- | --- |
| For plasmids construction |  |
| 2bUp1 | TCTC*GAGCTCCCCGGG*AAACGATAATTTAAGGTTCAGTTAAGGAAGTAATCGCG*GGATCC*TCTCTC^a^ |
| 2bUp2 | GAGAGA*GGATCC*CGCGATTACTTCCTTAACTGAACCTTAAATTATCGTTT*CCCGGGGAGCTC*GAGA^a^ |
| 2bUp6 | TC*GAGCTCCCCGGG*TGATAGCCCTGAGTTAGGA^b^ |
| 2bUp12 | TCTC*GGATCC*ATTATCGTTTAGATATTATATC^c^ |
| 2bUp14 | TCTC*CCCGGG*GACTTCGATGAGATTTTTC^d^ |
| 2bUp20 | TCTC*GGATCC*GTCCTGTGTTTTTTCTC^c^ |
|  |  |
| For qRT-PCR |  |
| GyrAgal-1 | GAGCGTCAGAGTCAAGCTAT |
| GyrAgal-2 | GCTTAACCTCATCCATCTCT |
| Spb1 | CCTGGGTCATCATTGCTAGT |
| Spb2 | CGATTACTATTCCGTGGGCA |
| ORF1 | GAACAGCAACTTCTTATTG |
| ORF2 | AATAGGTATCTGCAGCTAA |
| Gly1 | atggcacagacagatatct |
| Gly3 | CTTCCTCGGAGGTATTGAAC |
| Gly4 | AGGATAGCGCTGCTTGACAA |
| SAN0698-1 | CCAGTAGCTCAGTTTGCGAC |
| SAN0698-2 | CATTGTCACCAAGTTTAGCATAG |
|  |  |
| For primer extension |  |
| 2bEA1 | CTGTAATAGATTATGTCAC |
| 2bEA2 | TTCAATAAGAAGTTGCTG |
| 2bEA3 | CATAATTTCTAGCAAGTC |

^a^ In italics, *Sac*I, *Sma*I and *Bam*HI sites

^b^ In italics, *Sac*I and *Sma*I sites

^c^ In italics, *Bam*HI site

^d^ In italics, *Sma*I site
